# Meetings of Societies

**Published:** 1948

**Authors:** 


### Meetings of Societies

### Bristol Medico-Chirurgical Society

THE Annual General Meeting was held at the University on Wednesday, October 8th, 1947. Mr. Chitty introduced the new President, Dr. T. Milling, who gave his inaugural address on "**The Life of Dr. Jenner**" (p. 1). The Officers and Committee for 1947–48 (p. 33) were elected.

The Secretary's Report was adopted : 66 new members were elected, bringing the membership up to 310. A tribute was paid to deceased members, particularly Drs. Russell Cooper, Herapath and Bryan Adams. The work of the past session was reviewed.

The Treasurer's Report was received and approved. The question arose whether the Society could continue to work with the reduced subscription, adopted as a temporary measure in 1936, and it was proposed (and confirmed at the November meeting) that the annual subscription should again be one and a half guineas, as formerly. The future of the *Journal* was also discussed, and it was decided (November, confirmed December) that the subscription should be 16s. annually. The Secretary reminded members that the Library was open on Thursdays until 10.0 p.m.

The Editor's Report (p. 24) was read and adopted. The gist of this was embodied in the Editorial Note of our Autumn, 1947, issue (p. 85). The Editor reminded members that the apparently large accumulation at the end of 1946 was deceptive, since there was a considerable sum due to the *Journal* account for members' subscriptions which had not been paid over. Also the payments for 1946–47 included *six* issues of the *Journal* (four for 1946 and two for 1947) instead of the usual four. These expenses were due to overtaking arrears caused by wartime difficulties, and would not be recurrent.

---

The second meeting of the Society was held on November 12th. Dr. Coralie Rendle Short gave an account of her experiences, entitled "**Bullets and Babies in China,**" which we hope to publish.

---

The third meeting was held on December 10th at the University: a clinical meeting.

---

The fourth meeting was held on January 14th, 1948. Dr. Campbell described "**Recent Work on Disseminated Sclerosis,**" with especial reference to such trace elements as lead and copper, to the geographic distribution and to the possibility of infection.

---

The fifth meeting was held on February 18th, when Professor W. W. D. Thomson, of Belfast, gave a description of "**Cancer of the Bronchus,**" illustrated by numerous lantern slides.

